# Resveratrol Improves Glycemic Control in Type 2 Diabetic Obese Mice by Regulating Glucose Transporter Expression in Skeletal Muscle and Liver

**DOI:** 10.3390/molecules22071180

**Published:** 2017-07-14

**Authors:** Caio Y. Yonamine, Erika Pinheiro-Machado, Maria L. Michalani, Ana B. Alves-Wagner, João V. Esteves, Helayne S. Freitas, Ubiratan F. Machado

**Affiliations:** Department of Physiology and Biophysics, Institute of Biomedical Sciences, University of São Paulo, São Paulo, Brazil. Av. Prof. Lineu Prestes 1524, São Paulo 05508-900, Brazil; caioyogi@icb.usp.br (C.Y.Y.); erika.pinheiro.machado@usp.br (E.P.-M.); maria.michalani@usp.br (M.L.M.); anabarbara_ta@yahoo.com.br (A.B.A.-W.); joaovesteves@gmail.com (J.V.E.); hfreitas@icb.usp.br (H.S.F.)

**Keywords:** polyphenol, GLUT4, GLUT2, *Pck1*, SIRT1, pyruvate tolerance test

## Abstract

Insulin resistance participates in the glycaemic control disruption in type 2 diabetes mellitus (T2DM), by reducing muscle glucose influx and increasing liver glucose efflux. GLUT4 (*Slc2a4* gene) and GLUT2 (*Slc2a2* gene) proteins play a fundamental role in the muscle and liver glucose fluxes, respectively. Resveratrol is a polyphenol suggested to have an insulin sensitizer effect; however, this effect, and related mechanisms, have not been clearly demonstrated in T2DM. We hypothesized that resveratrol can improve glycaemic control by restoring GLUT4 and GLUT2 expression in muscle and liver. Mice were rendered obese T2DM in adult life by neonatal injection of monosodium glutamate. Then, T2DM mice were treated with resveratrol for 60 days or not. Glycaemic homeostasis, GLUT4, GLUT2, and SIRT1 (sirtuin 1) proteins (Western blotting); *Slc2a4*, *Slc2a2*, and *Pck1* (key gluconeogenic enzyme codifier) mRNAs (RT-qPCR); and hepatic glucose efflux were analysed. T2DM mice revealed: high plasma concentration of glucose, fructosamine, and insulin; insulin resistance (insulin tolerance test); decreased *Slc2a4*/GLUT4 content in gastrocnemius and increased *Slc2a2*/GLUT2 content in liver; and increased *Pck1* mRNA and gluconeogenic activity (pyruvate tolerance test) in liver. All alterations were restored by resveratrol treatment. Additionally, in both muscle and liver, resveratrol increased SIRT1 nuclear content, which must participate in gene expression regulations. In sum, the results indisputably reveals that resveratrol improves glycaemic control in T2DM, and that involves an increase in muscle *Slc2a4*/GLUT4 and a decrease in liver *Slc2a2*/GLUT2 expression. This study contributes to our understanding how resveratrol might be prescribed for T2DM according to the principles of evidence-based medicine.

## 1. Introduction

Diabetes mellitus (DM) is an epidemic metabolic disease, whose incidence is exponentially growing around the world [1]. Type 2 diabetes mellitus (T2DM) represents more than 90% of diabetic subjects, and has been related to obesity [2]. Insulin resistance plays a key role in the disruption of glycaemic homeostasis in T2DM, which leads to pancreatic beta cell failure [2]. Long-term evolution of T2DM is accompanied by the development of macro and microvascular diseases [3], which determine high morbidity and mortality. The continuous and constant improvement of glycaemic control is recognized as the best approach to reduce the development or progression of chronic complications in DM [4].

Since insulin resistance plays a vital role in the pathophysiology of T2DM, insulin sensitizer agents occupy the first place in the T2DM pharmacopeia, and new agents with this potential effect have been extensively investigated. Among several new compounds already investigated to treat T2DM, resveratrol has shown to be quite promising [5,6]. Resveratrol is a natural polyphenol widely found in several plants, mainly in grapes and blueberries [7]. Its biological effect is mainly related to the activation of a NAD^+^-dependent histone deacetylase sirtuin 1 (SIRT1) [5]. SIRT1 can be found in both cytosolic and nuclear compartments; in the latter, it can directly regulate gene expression [8].

Resveratrol has been reported to have a glycaemia-lowering effect in normal rats and mice [9,10], high-fat fed mice [11], and T2DM mice [12,13,14], as well as in non-diabetic obese [15] and in overweight T2DM humans [16,17]. However, most of these studies, especially in humans, reveal meagre results regarding glycaemic control, and drug-related mechanisms of action, which would strengthen resveratrol’s beneficial effect.

Glycaemic homeostasis results from an orchestrated regulation of several territorial glucose fluxes into and out of extracellular/blood compartments [18,19]. Some of these fluxes are highly variable and tightly regulated, thus altering glycaemia rapidly and prominently. This involves the skeletal muscle glucose uptake and the hepatic glucose outflow [18,19,20]. These fluxes are highly regulated by insulin, and insulin resistance is characterized by reduced skeletal muscle uptake and/or increased hepatic efflux of glucose, both concurring to increase plasma glucose concentration [18,19,20].

The glucose flow into or out of cells is processed by a variety of one, or more, glucose transporter proteins [21]. In skeletal muscle, glucose uptake is mediated by GLUT4 (solute carrier family 2, facilitated glucose transporter member 4) protein, codified by the *Slc2a4* (solute carrier family 2 member 4) gene [22]. GLUT4, stored in intracellular vesicles, can be rapidly translocated to the plasma membrane in response to insulin [22], and this mechanism is fundamental to the postprandial glycaemic regulation. Differently, in hepatocytes, the glucose flux is mediated by GLUT2 (solute carrier family 2, facilitated glucose transporter member 2) protein, codified by the *Slc2a2* (solute carrier family 2 member 2) gene [21]. The GLUT2, primarily located in hepatocyte plasma membrane, can transport glucose bidirectionally, and that depends on the substrate concentration gradient: in the postprandial state the glucose influx is favoured, whereas in the fasting state, increased intracellular glucose production favours the glucose efflux [20]. Thus, GLUT2-mediated glucose transport is not directly, but indirectly, regulated by insulin, according to the hormone regulation of the glucose concentration gradient. In postprandial state, high insulin levels stimulate hepatocyte glucose utilization, lowering intracellular concentration of glucose, thus generating a glucose influx gradient [20]. In the fasting state, low insulin levels release the gluconeogenic activity, thus generating intracellular glucose and a glucose efflux gradient [20].

As pointed out above, loss of glycaemic control in T2DM involves reduced muscle glucose influx and increased hepatic glucose efflux, which are directly related to reduced GLUT4 and increased GLUT2 expression, respectively [21,22]. In this context, we hypothesize that resveratrol could improve glycaemic control in T2DM, acting as an insulin sensitizer, and for that it must regulate GLUT4 and GLUT2 expressions, the molecular markers of glucose fluxes in muscle and liver.

## 2. Results

### 2.1. Resveratrol Restored Glycemic Homeostasis in T2DM Mice, without Attenuation of Body Weight Gain

**Figure 1:**
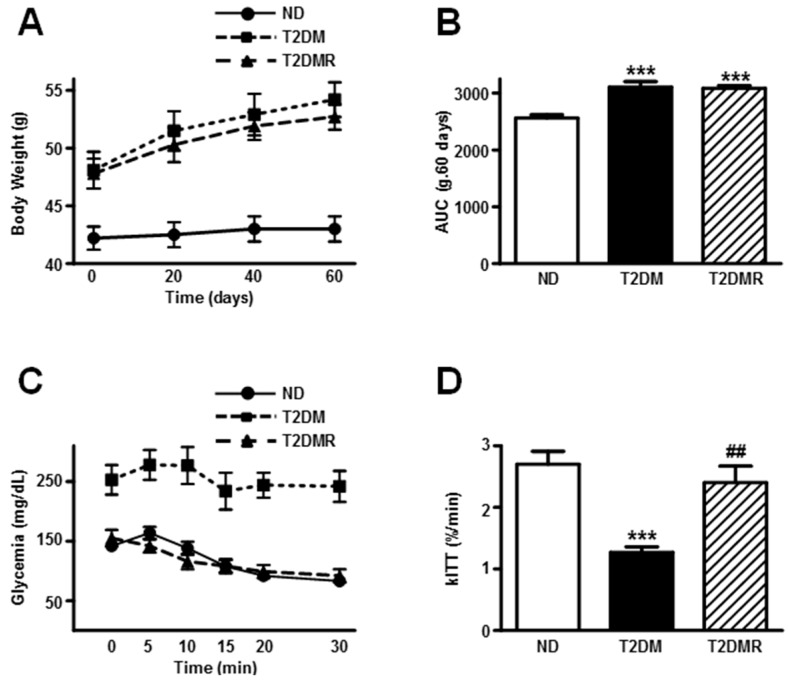
Resveratrol restored insulin resistance of T2DM mice, but did not alter obesity. Body weight evolution (**A**,**B**) and insulin sensitivity (**C**,**D**) were analysed in non-diabetic (ND), type 2 diabetic (T2DM) and resveratrol-treated type 2 diabetic (T2DMR) mice. (**A**) Body weight evolution; (**B**) area under the curve (AUC) of the body weight evolution during 60-day resveratrol treatment; (**C**) Blood glucose values; and (**D**) the blood glucose disappearance constant (kITT) during the insulin tolerance test (ITT). Data are expressed as mean ± SEM of 10 (body weight) or seven (ITT) animals, and were analysed by one-way ANOVA with the Newman-Keuls post-test. *** *p* < 0.001 vs. ND; ## *p* < 0.01 vs. T2DM.

At the age of 27 weeks, the T2DM mice were obese (Table 1), as evinced by elevated body weight and adipose tissue mass, as well as by increased Lee’s index value (*p* < 0.001 vs. ND). Conversely, the body length and gastrocnemius weight were decreased in T2DM mice (*p* < 0.001 and *p* < 0.01 vs. ND, respectively). The resveratrol treatment did not change the final data related to the obesity development (Table 1); however, the gastrocnemius mass was partially recovered (T2DMR vs. T2DM, *p* < 0.01). Furthermore, the body weight evolution during the 60-day resveratrol treatment (Figure 1A,B) also revealed an increased body weight gain in both T2DM and T2DMR mice, as compared to ND (*p* < 0.001).

Concerning the metabolic data, the T2DM mice presented higher plasma insulin, glucose, fructosamine, and triglycerides levels (Table 1), and the insulin tolerance test confirmed the insulin-resistant condition of these mice (Figure 1C,D). Resveratrol treatment reversed all metabolic data above to similar values of ND mice.

### 2.2. Resveratrol Increased Expression of Slc2a4/GLUT4 and Nuclear SIRT1 Protein Content in Gastrocnemius Muscle

**Figure 2:**
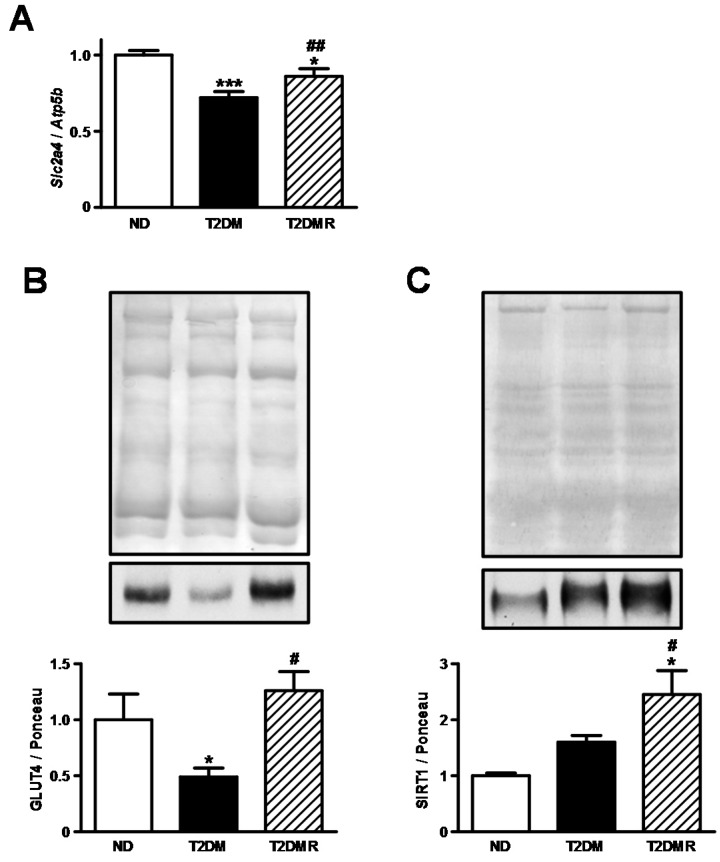
Resveratrol restored *Slc2a4* mRNA and GLUT4 protein expression in skeletal of T2DM mice. *Slc2a4* mRNA (**A**); GLUT4 protein (**B**), and nuclear SIRT1 protein (**C**) were analysed in gastrocnemius skeletal muscle of non-diabetic (ND), type 2 diabetic (T2DM), and resveratrol-treated type 2 diabetic (T2DMR) mice. In (**A**), *Slc2a4* mRNA was normalized by *Atp5b* mRNA (ATP synthase, H+ transporting, mitochondrial F1 complex, beta polypeptide); In (**B**,**C**), representative images of GLUT4 and SIRT1 proteins and respective Ponceau-stained membranes are shown. Data are expressed as mean ± SEM of 5–7 animals, and were analysed by one-way ANOVA with the Newman-Keuls post-test. * *p* < 0.05 and *** *p* < 0.001 vs. ND; # *p* < 0.05, and ## *p* < 0.01 vs. T2DM.

Aiming to address the involvement of skeletal muscle in the regulation of whole-body glucose homeostasis, we evaluated the *Slc2a4* mRNA and GLUT4 protein content in gastrocnemius. In the gastrocnemius of T2DM mice (Figure 2A,B), the mRNA of *Slc2a4* was reduced by 32% (*p* < 0.001 vs. ND) and the protein content of GLUT4 was reduced by 50% (*p* < 0.05 vs. ND). Resveratrol treatment partially restored the mRNA content, and completely restored the protein content (Figure 2A,B). Considering that SIRT1 is the main mediator of resveratrol effects, and plays a significant role in the regulation of gene expression, we evaluated the total SIRT1 content in the nuclear compartment. Although no differences were observed between ND and T2DM mice (Figure 2C), resveratrol treatment increased SIRT1 nuclear content by 126% (*p* < 0.05 vs. ND and T2DM).

### 2.3. Resveratrol Decreased Expression of Slc2a2/GLUT2 and Increased Nuclear SIRT1 Protein Content in Liver

**Figure 3:**
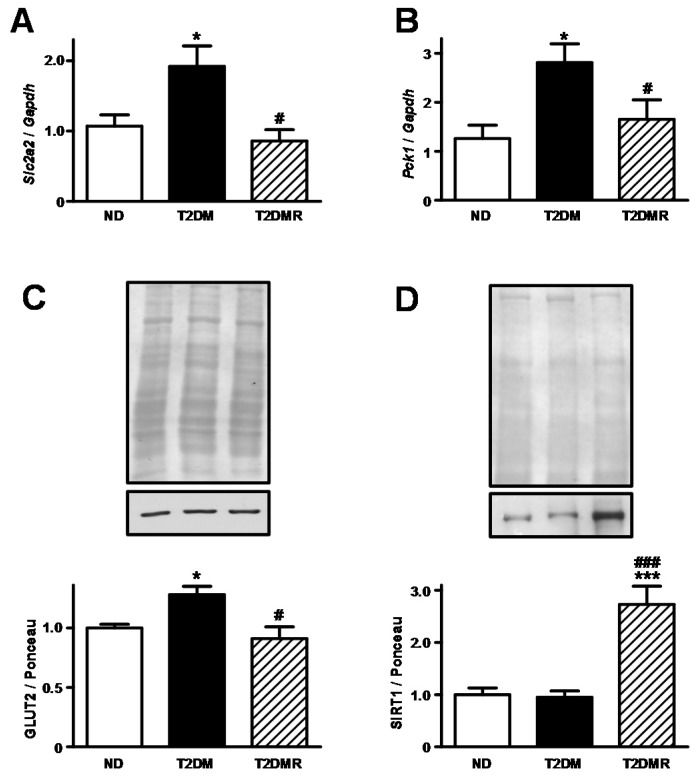
Resveratrol restored *Slc2a2* and *Pck1* mRNAs and GLUT2 protein expression in the liver of T2DM mice. *Slc2a4* mRNA (**A**), GLUT2 protein (**C**), *Pck1* mRNA (**B**), and nuclear SIRT1 protein (**D**) were analysed in the liver of non-diabetic (ND), type 2 diabetic (T2DM), and resveratrol-treated type 2 diabetic (T2DMR) mice. In (**A**,**B**), *Slc2a2* and *Pck1* mRNAs were normalized by *Gapdh* mRNA (glyceraldehyde-3-phosphate dehydrogenase); in (**C**,**D**), representative images of GLUT4 and SIRT1 proteins and respective Ponceau-stained membranes are shown. Data are expressed as mean ± SEM of 5–7 animals, and were analysed by one-way ANOVA with the Newman-Keuls post-test. * *p* < 0.05 and *** *p* < 0.001 vs. ND; # *p* < 0.05, and ### *p* < 0.001 vs. T2DM.

Aiming to address the role of hepatic territory in the regulation of whole-body glucose homeostasis, we evaluated the *Slc2a2* mRNA and GLUT2 protein content (Figure 3A,C). Under the T2DM condition, the *Slc2a2* mRNA and GLUT2 protein content increased by 90% and 27% (*p* < 0.05 vs. ND), respectively, and resveratrol treatment reversed these alterations. Nuclear SIRT1 in hepatic territory was unchanged comparing ND with T2DM mice (Figure 3D). However, resveratrol treatment in T2DM mice increased the nuclear SIRT1 by 173% (*p* < 0.001 vs. ND and T2DM).

**Figure 4:**
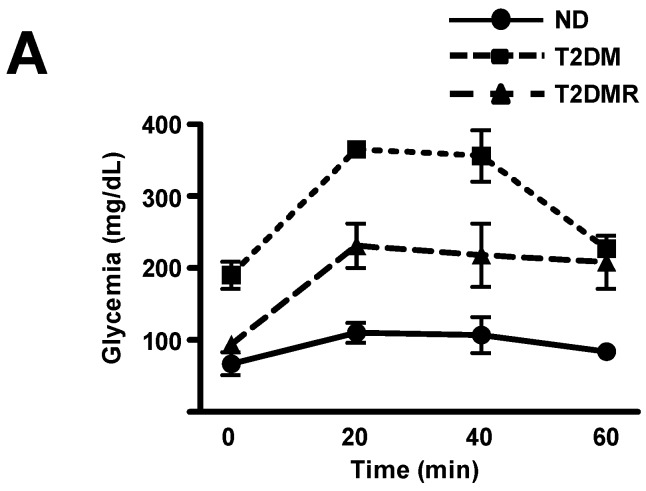

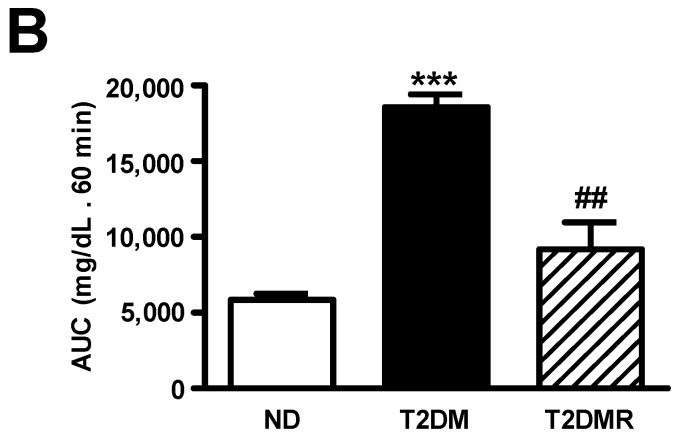
Resveratrol reduced in vivo gluconeogenic activity in T2DM mice. Blood glucose (**A**) and area under the curve (AUC) of blood evolution (**B**) during pyruvate tolerance test were analysed in non-diabetic (ND), type 2 diabetic (T2DM), and resveratrol-treated type 2 diabetic (T2DMR) mice. Data are expressed as mean ± SEM of 3 animals, and were analysed by one-way ANOVA with the Newman-Keuls post-test. *** *p* < 0.01 vs. ND; ## *p* < 0.05 vs. T2DM.

In order to predict the hepatic gluconeogenic activity, the expression of phosphoenolpyruvate carboxykinase 1 (*Pck1*) gene, which codifies the key gluconeogenic enzyme phosphoenolpyruvate carboxykinase (PEPCK), was measured (Figure 3B); the *Pck1* mRNA increased by 123% in T2DM (*p* < 0.05 vs. ND), and resveratrol reversed this effect (*p* < 0.05 vs. T2DM). To confirm the functional relevance of these data, i.e., the hepatic gluconeogenic activity, the pyruvate tolerance test was performed (Figure 4A,B). The results evinced that the glucose production in response to the pyruvate overload was three-fold higher in T2DM than in ND mice (*p* < 0.01), and resveratrol treatment reversed this data (*p* < 0.05 vs. T2DM).

### 2.4. Resveratrol Attenuates mRNA Content of Pro-Inflammatory Cytokines in White Adipose Tissue

**Figure 5:**
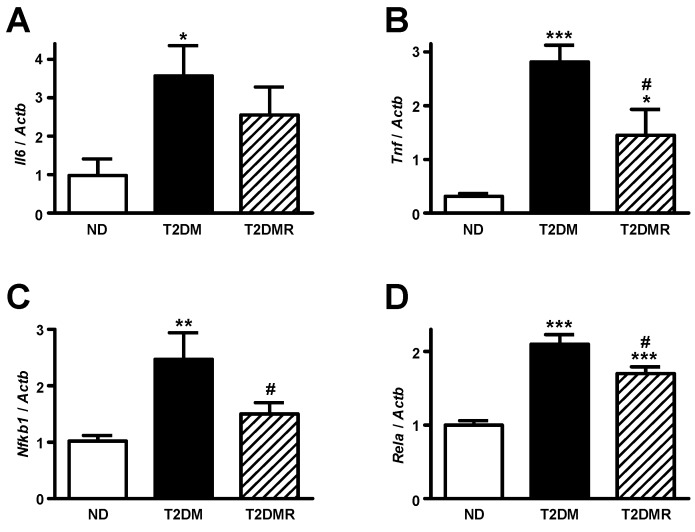
Resveratrol attenuated pro-inflammatory cytokine expression in epididymal adipose tissue of T2DM mice. Interleukin 6 (*Il6*, **A**); tumour necrosis factor (*Tnf*, **B**); nuclear factor kappa B subunit 1 (*Nfkb1*, **C**); and RELA proto-oncogene NF-κB subunit (*Rela*, **D**) mRNAs were measured in epididymal adipose tissue of non-diabetic (ND), type 2 diabetic (T2DM) and resveratrol-treated type 2 diabetic (T2DMR) mice. All target mRNAs were normalized by *Actb* mRNA (actin beta). Data are expressed as mean ± SEM of seven animals, and were analysed by one-way ANOVA with the Newman-Keuls post-test. * *p* < 0.05, ** *p* < 0.01 and *** *p* < 0.001 vs. ND; # *p* < 0.05 vs. T2DM.

Although no differences were observed in periepididymal adipose tissue weight after resveratrol treatment, we assessed the mRNA content of pro-inflammatory-related genes in this territory, considering that: (1) T2DM is associated to a chronic low-grade inflammation state; and (2) resveratrol has been described as having anti-inflammatory effects. In adipose tissue of T2DM mice (Figure 5A–D) the mRNA of *Tnf*, *Il6*, *Rela*, and *Nfkb1* was increased in comparison to ND mice (*p* < 0.05 to *p* < 0.001). The resveratrol treatment partially reversed the expression of *Tnf* and *Rela* (*p* < 0.05 vs. T2DM), and totally reversed the expression of *Nfkb1* (*p* < 0.05 vs. T2DM).

## 3. Discussion

The present study investigated the effectiveness of resveratrol on the glycaemic control in a model of obese T2DM mice. Nearly half a century ago, neonatal administration of MSG to mice was described as capable of inducing obesity in adult life, and that was associated to hypothalamic lesions [23]. Later on, after the participation of hepatic and muscular insulin resistance in the pathophysiology of T2DM was clear [24], our group observed that adult MSG mice develop hyperglycaemia with insulin resistance [25,26,27,28], thus characterizing an obese T2DM experimental model. Interestingly, the serious glycaemic impairment courses with hyperinsulinemia in T2DM mice, a profile not observed in humans, in whom the loss of glycaemic control only appears when plasma insulin levels start to decrease [24]. This difference must be a consequence of the high proliferative capacity of mice pancreatic islets.

As expected, the mice became severely obese, and developed a T2DM based on their high levels of plasma glucose and fructosamine. Furthermore, the mice were also extremely insulin resistant, as indicated by their hyperinsulinemia and low glucose decay during the insulin tolerance test. Importantly, the 60-day resveratrol treatment completely restored the glycaemic homeostasis of the obese T2DM mice, although they showed no weight loss in response to the treatment. An anti-hyperglycaemic effect was described in Zucker rats subjected to a similar treatment with resveratrol [29], although hyperglycaemia was not so high as in the present study. On the other hand, the anti-obesity effects of resveratrol are variable, probably due to variable experimental models and resveratrol doses and times of treatment [30,31,32,33]. It is important to highlight that, in the present obese T2DM mice, resveratrol recovered the glycaemic homeostasis completely, without attenuation of body or fat mass, pointing out that the metabolic effects are unrelated to weight loss.

The pathophysiology of insulin resistance, related to the development and progression of T2DM, involves reduced skeletal muscle glucose uptake and/or increased hepatic glucose outflow. Due to this, the present study evaluated the expression of glucose transporter isoforms GLUT4 and GLUT2, which are molecular markers of muscle influx and liver efflux of glucose, respectively.

Skeletal muscle accounts for approximately 75% of whole-body insulin-stimulated glucose disposal [34,35], which explains the central role of muscle in the insulin resistance induced impairment of glycemic homeostasis, especially in the postprandial period. The present results clearly show that the *Slc2a4* gene and GLUT4 protein expression, which are decreased in T2DM, recovered in response to resveratrol, and that can explain previous considerations that resveratrol can improve muscle glucose uptake in high-fat-fed rats [36]. GLUT4 was described to increase just in one db/db mouse treated with resveratrol [10], and the effects of resveratrol on glycaemic homeostasis in that study were not clearly convincing. Additionally, increased insulin-induced GLUT4 translocation to the plasma membrane, but not total protein expression, was suggested in muscles from insulin-resistant rats treated with resveratrol [37]; however, this effect was not confirmed in isolated muscle cells [38].

Once the present data revealed, for the first time, that resveratrol increases the GLUT4 protein expression in muscle from T2DM mice, by a mechanism that involves enhanced *Slc2a4* gene expression, we logically thought this would be related to SIRT1-mediated intranuclear effect of resveratrol. Indeed, resveratrol increased the nuclear SIRT1 content in muscle. Several studies have reported that SIRT1 activation by resveratrol leads to deacetylation of the PPAR-G-coactivator 1 (PGC1), enhancing its activity [30,39]. Importantly, PGC1 is a potent enhancer of the *Slc2a4* gene transcription [40]. Furthermore, we cannot exclude a deacetylase effect of SIRT1 directly into the *Slc2a4* gene promoter, impairing the activity of enhancer factors. Together, these mechanisms can explain a SIRT1-mediated resveratrol-induced improvement of *Slc2a4*/GLUT4 expression.

The participation of the liver in the impairment of glycaemic control in T2DM has been related to the increased GLUT2 expression. However, to allow greater glucose efflux, it is necessary to increase the glucose production in the hepatocyte, which is guaranteed by increased gluconeogenic activity [41,42]. The present data reveal that T2DM increased the expression of *Slc2a2* and *Pck1* genes, which codify the glucose transporter and the key gluconeogenic enzyme, respectively. These molecular markers indicated increased hepatic glucose outflow, which was observed in response to an overload of the gluconeogenic precursor pyruvate. All these alterations are reversed by resveratrol, revealing a reduction in the glucose hepatic outflow, which must have contributed to improving the glycaemic control.

The results demonstrate that resveratrol treatment of T2DM mice increased nuclear SIRT1 content also in the liver, which was previously observed in insulin-treated T1DM rat, is also accompanied by a reduction in *Slc2a2* gene expression [43]. Increased expression of *Slc2a2*/GLUT2 in the liver of T1DM diabetic rats was related to the enhanced activity of hepatocyte nuclear factor 1 alpha (HNF1alpha) [44]. Regarding that, in primary hepatocytes, the overexpression of SIRT1 was reported to decrease HNF1alpha transcriptional activity, with a consequent repression of its target gene *Slc2a2* [45]. However, investigation of the deletion of SIRT1 in hepatocytes also showed a reduction in *Slc2a2* expression, again through the HNF1alpha-mediated manner [46]. Moreover, in response to SIRT1 overexpression, *Pck1* expression was reported to be upregulated [45], although other studies have described that SIRT1 regulates gluconeogenesis negatively by repressing *Pck1* [39,47], as observed here.

Obesity, insulin resistance, and T2DM are closely associated with chronic inflammation [48], and that seems to primarily affect the white adipose tissue [49]. In this process, the adipose tissue increases pro-inflammatory cytokine production, and exports these cytokines to other territories [50]. Since anti-inflammatory effects have been attributed to resveratrol [51], that could be an additional mechanism involved in its beneficial effect. The results confirmed high inflammatory activity in adipose tissue of T2DM mice, as evinced by the increased expression of *Tnf*, *Il6*, *Rela*, and *Nfkb1* genes. Additionally, resveratrol treatment reduced the expression of these inflammatory genes, except *Il6*. Considering those results, we can suggest that resveratrol triggered an anti-inflammatory activity in adipose tissue, which might also participate in its beneficial effect upon the glycaemic homeostasis in T2DM mice.

In view of the present results, we conclude that resveratrol recovers glycaemic homeostasis in T2DM mice, and that involves increased *Slc2a4*/GLUT4 expression in muscle and decreased *Slc2a2*/GLUT2 expression in the liver, which may be related to increased nuclear SIRT1 content. This study contributes to our understanding of how resveratrol might be prescribed for T2DM according to the principles of evidence-based medicine.

## 4. Materials and Methods

### 4.1. Animal Treatment

Obesity and type 2 diabetes (T2DM) was carried out by neonatal subcutaneous injections of monosodium glutamate (2 mg/g body weight) in male offspring mice (CD1) from day one to day five after birth [25]. Control mice received subcutaneous injections of 0.9% NaCl. Animals were weaned, fed standard rodent chow, and allowed access to water ad libitum until 19 weeks old, when half of the MSG animals started to be treated with resveratrol (Sigma Chemical Co., St. Louis, MO, USA), given in water, in a dose of 30 mg/Kg of body weight, for 60 days. According to the water intake, the resveratrol concentration in the water was weekly calculated, to preserve the prescribed dose. Then, three groups of animals were investigated: non-diabetic (ND), obese type 2 diabetic (T2DM), and T2DM treated with resveratrol (T2DMR). Food and water consumption was monitored twice a week. All procedures performed in this study were approved by the Ethical Committee for Animal Research of the Institute of Biomedical Sciences, University of São Paulo (004/2015).

### 4.2. Sample Collection

At the end of treatment (27-week-old mice), the animals were killed between 8 a.m. and 10 a.m. (after 4 h of food deprivation) under anesthesia (60 mg/kg body weight sodium pentobarbital, intraperitoneally). Firstly, the naso-anal length was measured to estimate the obesity degree by the Lee´s index (body weight (g)^1/3^/naso-anal length (cm)) × 100. After that, gastrocnemius muscle, epididymal white adipose tissue, and liver were sampled and stored at −70 °C for further analysis. After liver sampling, blood was collected from the heart left ventricle, centrifuged at 2000× *g* for 10 min at 4 °C, and the plasma was stored for glucose, fructosamine, insulin, and triglyceride concentration analyses.

### 4.3. Metabolic Analysis

The concentrations of plasma glucose, triglycerides, and fructosamine were determined by colorimetric assay and performed according to manufacturer recommendations (Labtest Diagnóstica SA, Lagoa Santa, MG, Brasil). The quantification of plasma insulin was determined by ELISA (enzyme linked immunonosorbent assay) and performed according to manufacturer recommendations (Cat. #EZRMI-13K, EMD Millipore Corporation, St. Charles, MO, USA).

**Insulin tolerance test** (**ITT**). Some animals were subjected to the ITT. Mice were food deprived for 4 h, and the test was performed at 10 a.m., under non-anesthetized condition. Tail blood was collected for determination of basal glycemia (0 min), then insulin was injected intraperitoneally (1 U/kg body weight), and tail blood was collected at 5, 10, 15, 20, and 30 min. Insulin sensitivity was analyzed by measuring the glucose disappearance constant (kITT), based on the linear regression of the Napierian logarithm of the glycemic values obtained from 5 to 30 min of the test [52].

**Pyruvate tolerance test** (**PTT**). Additional animals were subjected to the PTT. For this test, mice were food deprived for 12 h, and the test performed at 8 a.m. Anesthetized animals (60 mg/kg body weight sodium pentobarbital, intraperitoneally) were injected with pyruvate solution (0.25 g/mL), at a dose of 2 g/kg, intraperitoneally. Glycaemia was measured in tail blood at 0 (basal), 15, 30, and 60 min after the pyruvate injection. The area under the curve of glycemia after a pyruvate load was calculated by using GraphPad Prism version 5.01 (GraphPad Software, San Diego, CA, USA), and represents the hepatic glucose production.

**Western blotting analyses of GLUT4**, **GLUT2, and SIRT1**. Muscle and liver samples were processed for GLUT4 and GLUT2, respectively, as previously described [43]. From muscle, a total cellular protein fraction was obtained, and from liver, a plasma membrane protein-enriched fraction was obtained. For SIRT1 analyses, nuclear proteins were extracted from both muscle and liver as described by Andrews and Faller [53]. Enrichment in nuclear protein was confirmed by measurement of the nuclear marker histone 1. Equal amounts of protein (30 to 100 μg, depending on the sample) were electrophoresed, transferred to a nitrocellulose membrane, and immunoblotted with anti-GLUT4 (EMD Millipore, Billerica, MA, USA, #07-1404), anti-GLUT2 (EMD Millipore, #07-1402), or anti-SIRT1 (Cell Signaling Technology, Danvers, MA, USA, mAB#8469) antibodies. The secondary conjugated antibody was used following specifications, and an enhanced chemiluminescence (ECL) procedure was performed. The optical densitometry of the blots and of the respective lanes, stained by Ponceau, was analyzed using Image J software (National Institutes of Health, Bethesda, MD, USA). The Ponceau-stained lane was used as loading control for normalization of the results [54]. The results were expressed as arbitrary units, related to the mean of the controls, which was set as 1.0.

**RT-qPCR analyses of *Slc2a4***, ***Slc2a2*, and *Pck1***. Gastrocnemius, periepididymal adipose tissue, and liver (100 mg) samples were used to obtain total RNA, by the TRIzol^®^ method, according to the manufacturer’s instructions (Invitrogen, Carlsbad, CA, USA). Nanodrop 2000 (Thermo Scientific, Waltham, MA, USA) was used to quantify total RNA in each sample. One µg of total RNA was used to perform the reverse transcriptase (RT) reaction, by adding oligo dT (100 µg/mL), 10 mM of each dNTP, 5X first-strand buffer, and 2 μL (200 U/μL) of M-MLV reverse transcriptase (Promega, Madison, WI, USA). The RT reaction was performed at 65 °C for 10 min, 37 °C for 60 min, and 95 °C for 10 min. The qPCR amplification of genes analyzed in muscle and liver was performed using a Taqman^®^ PCR master mix kit (Applied Biosystems Inc., Foster City, CA, USA), and carried out in a StepOne Plus instrument (Applied Biosystems Inc.). The qPCR amplification of genes analyzed in adipose tissue was performed using SYBR^®^ Green Real-Time PCR Master Mix (Thermo Fisher Scientific, Waltham, MA, USA) and carried out in a StepOne Plus instrument (Applied Biosystem Inc.). Several reference genes were tested for their stability using the RefFinder software, and final choices were: ATP synthase, H+ transporting, mitochondrial F1 complex, beta polypeptide (*Atp5b*) for skeletal muscle; glyceraldehyde-3-phosphate dehydrogenase (*Gapdh*) for liver, and actin beta (*Actb*) for adipose tissue. Details of the Taqman^®^ and SYBR^®^ gene expression assays used for qPCR are described in Table 2. Gene expression results were calculated based on the 2^–ΔΔCt^ method.

### 4.4. Statistical Analysis

Results were expressed as the mean ± standard error of the mean (SEM). The means were compared by one-way analysis of variance (ANOVA), followed by Newman-Keuls post-test, after checking the variances by Bartlett’s test. Body weight gain during the treatment, and glycaemia variation during the pyruvate tolerance test, were analyzed by comparing the means of individual values of the area under the curve. Insulin sensitivity in the insulin tolerance test was analyzed by comparing the means of the individual values of the glucose disappearance constant (kITT). Differences were considered statistically significant at *p* ≤ 0.05.

## Figures and Tables

**Table 1:** Morphometric and metabolic characteristics of the non-diabetic (ND), type 2 diabetic (T2DM), and resveratrol-treated type 2 diabetic (T2DMR) mice.

	ND	T2DM	T2DMR
Body weight (g)	43.0 ± 1.06	54.2 ± 1.48 ***	52.7 ± 1.09 ***
Length (cm)	10.70 ± 0.17	9.20 ± 0.13 ***	9.46 ± 0.13 ***
Lee index	32.7 ± 0.46	40.3 ± 0.52 ***	39.2 ± 0.40 ***
Adipose tissue weight (g)	1.01 ± 0.09	2.07 ± 0.20 ***	2.39 ± 0.16 ***
Skeletal muscle weight (mg)	156.7 ± 6.7	110.0 ± 8.2 **	136.6 ± 12.5 **^,^^##^
Plasma insulin (ng/mL)	0.73 ± 0.28	5.34 ± 0.90 ***	1.63 ± 0.73 ^##^
Plasma glucose (mg/dL)	132.0 ± 14.0	243.6 ± 18.9 ***	133.9 ± 8.5 ^###^
Plasma fructosamine (µmol/L)	131.8 ± 4.90	202.0 ± 25.8 *	118.7 ±12.8 ^#^
Plasma triglycerides (mg/dL)	100.0 ± 11.5	225.6 ± 38.2 *	128.80 ± 33.2 ^#^

Adipose tissue, epididymal white adipose tissue; skeletal muscle, gastrocnemius. Data are expressed as mean ± SEM of 10 animals, and were compared by one-way ANOVA, Student Newman-Keuls post-test. * *p* < 0.05, ** *p* < 0.01 and *** *p* < 0.001 vs. ND; ^#^ *p* < 0.05, ^##^ *p* < 0.01, and ^###^ *p* < 0.001 vs. T2DM.

**Table 2:** Details of the Taqman^®^ and SYBR^®^ gene expression assays used for real-time polymerase chain reaction (qPCR).

Gene	Primers Sequence	Dye	Assay ID
*Slc2a4*	Inventoried	FAM	Mm01245502_m1
*Slc2a2*	Inventoried	FAM	Mm00446229_m1
*Gapdh*	Inventoried	VIC	Mm99999915_g1
*Pck1*	Inventoried	FAM	Mm01247058_m1
*Atp5b*	Inventoried	FAM	Mm00443967_g1
*Tnf*	F: GAACTGGCAGAAGAGGCACT	SYBR	
	R: GGTCTGGGCCATAGAACTGA		
*Il6*	F: CCGGAGAGGAGACTTCACAG	SYBR	
	R: TCCAGTTTGGTAGCATCCATC		
*Actb*	F: ACTGGGACGACATGGAGAAG	SYBR	
	R: GGGGTGTTGAAGGTCTCAAA		
*Rela*	F: GCAAGGGCATTATCGACTCT	SYBR	
	R: CATAACGTTGCAGGAAGCTG		
*Nfkb1*	F: CTGACCTGAGCCTTCTGGAC	SYBR	
	R: GCAGGCTATTGCTCATCACA		

Assay ID identifies the specific TaqMan^®^ RNA Assay (Applied Biosystems Inc., USA); SYBR^®^ refers to SYBR^®^ Green Real-Time PCR Master Mix (Termo Fisher Scientific). *Slc2a4*, solute carrier family 2 member 4; *Slc2a2*, solute carrier family 2 member 2; *Gapdh*, glyceraldehyde-3-phosphate dehydrogenase; *pck1*, phosphoenolpyruvate carboxykinase 1; *Atp5b*, ATP synthase, H+ transporting, mitochondrial F1 complex, beta polypeptide; *Tnf*, tumor necrosis factor; *Il6*, interleukin 6; *Actb*, actin beta; *Rela*, RELA proto-oncogene, NF-κB subunit; *Nfkb1*, nuclear factor kappa B subunit 1.

## References

[1] International Diabetes Federation IDF Atlas-7th Edition. http://www.diabetesatlas.org.

[2] Thomas C.C., Philipson L.H. (2015). Update on diabetes classification. Med. Clin. N. Am..

[3] Orasanu G., Plutzky J. (2009). The pathologic continuum of diabetic vascular disease. J. Am. Coll. Cardiol..

[4] American Diabetes Association (2016). Standards of Medical Care in Diabetes–2016. Diabetes Care.

[5] Szkudelski T., Szkudelska K. (2015). Resveratrol and diabetes: From animal to human studies. Biochim. Biophys. Acta.

[6] Aguirre L., Fernández-Quintela A., Arias N., Portillo M.P. (2014). Resveratrol: Anti-obesity mechanisms of action. Molecules.

[7] Kasiotis K.M., Pratsinis H., Kletsas D., Haroutounian S.A. (2013). Resveratrol and related stilbenes: Their anti-aging and anti-angiogenic properties. Food Chem. Toxicol..

[8] Tanno M., Sakamoto J., Miura T., Shimamoto K., Horio Y. (2007). Nucleocytoplasmic shuttling of the NAD+-dependent histone deacetylase SIRT1. J. Biol. Chem..

[9] Chi T.C., Chen W.P., Chi T.L., Kuo T.F., Lee S.S., Cheng J.T., Su M.J. (2007). Phosphatidylinositol-3-kinase is involved in the antihyperglycemic effect induced by resveratrol in streptozotocin-induced diabetic rats. Life Sci..

[10] Do G.M., Jung U.J., Park H.J., Kwon E.Y., Jeon S.M., McGregor R.A., Choi M.S. (2012). Resveratrol ameliorates diabetes-related metabolic changes via activation of AMP-activated protein kinase and its downstream targets in *db*/*db* mice. Mol. Nutr. Food Res..

[11] Zhang J., Chen L., Zheng J., Zeng T., Li H., Xiao H., Deng X., Hu X. (2012). The protective effect of resveratrol on islet insulin secretion and morphology in mice on a high-fat diet. Diabetes Res. Clin. Pract..

[12] Minakawa M., Miura Y., Yagasaki K. (2012). Piceatannol, a resveratrol derivative, promotes glucose uptake through glucose transporter 4 translocation to plasma membrane in L6 myocytes and suppresses blood glucose levels in type 2 diabetic model *db*/*db* mice. Biochem. Biophys. Res. Commun..

[13] Uchida-Maruki H., Inagaki H., Ito R., Kurita I., Sai M., Ito T. (2015). Piceatannol lowers the blood glucose level in diabetic mice. Biol. Pharm. Bull..

[14] Chen S., Li J., Zhang Z., Li W., Sun Y., Zhang Q., Feng X., Zhu W. (2012). Effects of resveratrol on the amelioration of insulin resistance in KKAy mice. Can. J. Physiol. Pharmacol..

[15] Timmers S., Konings E., Bilet L., Houtkooper R.H., van de Weijer T., Goossens G.H., Hoeks J., van der Krieken S., Ryu D., Kersten S. (2011). Calorie restriction-like effects of 30 days of resveratrol supplementation on energy metabolism and metabolic profile in obese humans. Cell Metab..

[16] Bhatt J.K., Thomas S., Nanjan M.J. (2012). Resveratrol supplementation improves glycemic control in type 2 diabetes mellitus. Nutr. Res..

[17] Movahed A., Nabipour I., Lieben Louis X., Thandapilly S.J., Yu L., Kalantarhormozi M., Rekabpour S.J., Netticadan T. (2013). Antihyperglycemic effects of short term resveratrol supplementation in type 2 diabetic patients. Evid. Based Complement. Altern. Med..

[18] DeFronzo R.A. (2004). Pathogenesis of type 2 diabetes mellitus. Med. Clin. N. Am..

[19] Kahn S.E., Cooper M.E., Del Prato S. (2014). Pathophysiology and treatment of type 2 diabetes: Perspectives on the past, present, and future. Lancet.

[20] Thorens B. (2015). GLUT2, glucose sensing and glucose homeostasis. Diabetologia.

[21] Thorens B., Mueckler M. (2010). Glucose transporters in the 21st Century. Am. J. Physiol. Endocrinol. Metab..

[22] Corrêa-Giannella M.L., Machado U.F. (2013). *SLC2A4* gene: A promising target for pharmacogenomics of insulin resistance. Pharmacogenomics.

[23] Olney J.W. (1969). Brain lesions, obesity, and other disturbances in mice treated with monosodium glutamate. Science.

[24] DeFronzo R.A. (1988). Lilly lecture 1987. The triumvirate: Beta-cell, muscle, liver. A collusion responsible for NIDDM. Diabetes.

[25] Machado U.F., Shimizu I., Saito M. (1994). Reduced content and preserved translocation of glucose transporter (GLUT 4) in white adipose tissue of obese mice. Physiol. Behav..

[26] Machado U.F., Shimizu Y., Saito M. (1993). Decreased glucose transporter (GLUT 4) content in insulin-sensitive tissues of obese aurothioglucose- and monosodium glutamate-treated mice. Horm. Metab. Res..

[27] Papa P.C., Seraphim P.M., Machado U.F. (1997). Loss of weight restores GLUT 4 content in insulin-sensitive tissues of monosodium glutamate-treated obese mice. Int. J. Obes. Relat. Metab. Disord..

[28] De Carvalho Papa P., Vargas A.M., da Silva J.L., Nunes M.T., Machado U.F. (2002). GLUT4 protein is differently modulated during development of obesity in monosodium glutamate-treated mice. Life Sci..

[29] Rivera L., Morón R., Zarzuelo A., Galisteo M. (2009). Long-term resveratrol administration reduces metabolic disturbances and lowers blood pressure in obese Zucker rats. Biochem. Pharmacol..

[30] Baur J.A., Pearson K.J., Price N.L., Jamieson H.A., Lerin C., Kalra A., Prabhu V.V., Allard J.S., Lopez-Lluch G., Lewis K. (2006). Resveratrol improves health and survival of mice on a high-calorie diet. Nature.

[31] Kim S., Jin Y., Choi Y., Park T. (2011). Resveratrol exerts anti-obesity effects via mechanisms involving down-regulation of adipogenic and inflammatory processes in mice. Biochem. Pharmacol..

[32] Lagouge M., Argmann C., Gerhart-Hines Z., Meziane H., Lerin C., Daussin F., Messadeq N., Milne J., Lambert P., Elliott P. (2006). Resveratrol improves mitochondrial function and protects against metabolic disease by activating SIRT1 and PGC-1alpha. Cell.

[33] Tauriainen E., Luostarinen M., Martonen E., Finckenberg P., Kovalainen M., Huotari A., Herzig K.H., Lecklin A., Mervaala E. (2011). Distinct effects of calorie restriction and resveratrol on diet-induced obesity and Fatty liver formation. J. Nutr. Metab..

[34] Koistinen H.A., Zierath J.R. (2002). Regulation of glucose transport in human skeletal muscle. Ann. Med..

[35] Zierath J.R., Krook A., Wallberg-Henriksson H. (2000). Insulin action and insulin resistance in human skeletal muscle. Diabetologia.

[36] Chen L.L., Zhang H.H., Zheng J., Hu X., Kong W., Hu D., Wang S.X., Zhang P. (2011). Resveratrol attenuates high-fat diet-induced insulin resistance by influencing skeletal muscle lipid transport and subsarcolemmal mitochondrial β-oxidation. Metabolism.

[37] Tan Z., Zhou L.J., Mu P.W., Liu S.P., Chen S.J., Fu X.D., Wang T.H. (2012). Caveolin-3 is involved in the protection of resveratrol against high-fat-diet-induced insulin resistance by promoting GLUT4 translocation to the plasma membrane in skeletal muscle of ovariectomized rats. J. Nutr. Biochem..

[38] Breen D.M., Sanli T., Giacca A., Tsiani E. (2008). Stimulation of muscle cell glucose uptake by resveratrol through sirtuins and AMPK. Biochem. Biophys. Res. Commun..

[39] Kitada M., Koya D. (2013). SIRT1 in Type 2 Diabetes: Mechanisms and Therapeutic Potential. Diabetes Metab. J..

[40] Michael L.F., Wu Z., Cheatham R.B., Puigserver P., Adelmant G., Lehman J.J., Kelly D.P., Spiegelman B.M. (2001). Restoration of insulin-sensitive glucose transporter (GLUT4) gene expression in muscle cells by the transcriptional coactivator PGC-1. Proc. Natl. Acad. Sci. USA.

[41] DeFronzo R.A. (2010). Current issues in the treatment of type 2 diabetes. Overview of newer agents: where treatment is going. Am. J. Med..

[42] Beale E.G., Harvey B.J., Forest C. (2007). PCK1 and PCK2 as candidate diabetes and obesity genes. Cell Biochem. Biophys..

[43] Yonamine C.Y., Pinheiro-Machado E., Michalani M.L., Freitas H.S., Okamoto M.M., Corrêa-Giannella M.L., Machado U.F. (2016). Resveratrol improves glycemic control in insulin-treated diabetic rats: Participation of the hepatic territory. Nutr. Metab. (Lond).

[44] David-Silva A., Freitas H.S., Okamoto M.M., Sabino-Silva R., Schaan B.D., Machado U.F. (2013). Hepatocyte nuclear factors 1α/4α and forkhead box A2 regulate the solute carrier 2A2 (*Slc2a2*) gene expression in the liver and kidney of diabetic rats. Life Sci..

[45] Grimm A.A., Brace C.S., Wang T., Stormo G.D., Imai S. (2011). A nutrient-sensitive interaction between Sirt1 and HNF-1α regulates Crp expression. Aging Cell.

[46] Purushotham A., Xu Q., Lu J., Foley J.F., Yan X., Kim D.H., Kemper J.K., Li X. (2012). Hepatic deletion of SIRT1 decreases hepatocyte nuclear factor 1α/farnesoid X receptor signaling and induces formation of cholesterol gallstones in mice. Mol. Cell. Biol..

[47] Wang R.H., Kim H.S., Xiao C., Xu X., Gavrilova O., Deng C.X. (2011). Hepatic Sirt1 deficiency in mice impairs mTorc2/Akt signaling and results in hyperglycemia, oxidative damage, and insulin resistance. J. Clin. Investig..

[48] Hotamisligil G.S. (2006). Inflammation and metabolic disorders. Nature.

[49] Lee B.C., Lee J. (2014). Cellular and molecular players in adipose tissue inflammation in the development of obesity-induced insulin resistance. Biochim. Biophys. Acta.

[50] Tishinsky J.M., De Boer A.A., Dyck D.J., Robinson L.E. (2014). Modulation of visceral fat adipokine secretion by dietary fatty acids and ensuing changes in skeletal muscle inflammation. Appl. Physiol. Nutr. Metab..

[51] Inoue H., Nakata R. (2015). Resveratrol Targets in Inflammation. Endocr. Metab. Immune Disord. Drug Targets.

[52] Mori R.C., Hirabara S.M., Hirata A.E., Okamoto M.M., Machado U.F. (2008). Glimepiride as insulin sensitizer: Increased liver and muscle responses to insulin. Diabetes Obes. Metab..

[53] Andrews N.C., Faller D.V. (1991). A rapid micropreparation technique for extraction of DNA binding proteins from limiting numbers of mammalian cells. Nucleic Acids Res..

[54] Romero-Calvo I., Ocón B., Martínez-Moya P., Suárez M.D., Zarzuelo A., Martínez-Augustin O., De Medina F.S. (2010). Reversible Ponceau staining as a loading control alternative to actin in Western blots. Anal. Biochem..

